# Death of Dr. Jerome Cochran

**Published:** 1896-09

**Authors:** 


THE MEDICAL PRESS announce the death, in Alabama, of Dr. Jerome Cochran. He died on the 17th of August, ult., after an invalidism, we learn, of many years. Dr. Cochran's death is a serious loss to the medical profession, not only of Alabama, but of the South. His work in organizing the State Board of Health of Alabama, and of getting it incorporated into the laws of the State so as to give it power to legislate on medical matters, will stand as an enduring monument not only to his ability as a medical politician, but to his earnestness as a devotee to his profession. Dr. Cochran was distinguished, further, by his work in connection with yellow fever; he being regarded as an authority on the disease, and an expert in its diagnosis and treatment. He was a member of the Yellow Fever Commission of 1878, appointed by Congress to report upon the epidemic of that year.

---

FROM ALL OVER TEXAS come reports and complaints to the TEXAS MEDICAL JOURNAL that the law relating to the practice of medicine—as lax as it is—is being violated, or disregarded, with impunity. Men well known to the profession; some even, who hold, or have held, high government positions, are found, upon inquiry, to not have complied with the simple requirement of registering their diplomas, if they have any; and instances have come to our knowledge, where men more or less prominent in the social and political world, have nothing on record where they live, but a temporary certificate from a single member of some board. In several instances, we are informed, efforts have been made to get district attorneys to look into the matter; but they seem to be very reluctant to do so; and these men, openly violating a law of the State, go unpunished.

The JOURNAL has taken it upon itself to call the attention of the Attorney-General to the subject, and to ask that he instruct all county and district judges to charge the grand jury to investigate the matter. We can give him some pointers, if he will accept them.

THE grand jury at Orange and Beaumont failed to indict two parties there practicing on Texas Health College diplomas; because, it is alleged, they have no proof that no such college exists, and there is no fund available for the purpose of securing such proof. We call attention to a letter on the subject in this issue, from one of our subscribers at Beaumont. In reply to the objection, we suggest, that if any physician at Beaumont or Orange will write to Dr. T. J. Smith, at Mound, Texas, or to Dr. J. E. Webb, at Trenton, Texas, either of these gentlemen will forward affidavits of citizens of Mound, free of charge, that there is not, and never has been, such an institution there as the "Texas Health College," or any other medical college.

